# Editorial: Clinical prediction models in cancer through bioinformatics

**DOI:** 10.3389/fbinf.2026.1902687

**Published:** 2026-06-19

**Authors:** Wenlin Yang, Jigang Wang, Nikee Awasthee, Ragini Singh

**Affiliations:** 1 Department of Physiology and Aging, College of Medicine, University of Florida, Gainesville, FL, United States; 2 Department of Pathology, The Affiliated Hospital of Qingdao University, Shandong, China; 3 Genetics Branch, National Cancer Institute, NIH, Bethesda, MD, United States

**Keywords:** artificial intelligence, bioinformatics, clinical prediction models (CPMs), machine learning, omics

The rapid advancement of bioinformatics, artificial intelligence (AI), and high-throughput omics technologies has fundamentally reshaped modern cancer research and precision oncology. Increasingly, computational approaches are being integrated into clinical oncology to improve diagnostic accuracy, prognostic assessment, therapeutic response prediction, complication risk estimation, and individualized patient stratification. Within this evolving landscape, the Research Topic “Clinical Prediction Models in Cancer Through Bioinformatics” was established to provide an interdisciplinary platform for studies exploring innovative bioinformatics-driven and AI-assisted predictive modeling strategies in oncology.

This Research Topic ultimately collected 25 articles spanning multiple cancer types, computational methodologies, and translational applications. The published studies collectively demonstrate the rapidly expanding role of genomics, transcriptomics, proteomics, metabolomics, imaging, immunological profiling, and clinicopathological data integration in the development of clinically relevant prediction models. Importantly, the collection highlights not only methodological innovation, but also the increasing emphasis on interpretability, external validation, and real-world translational potential.

A central theme emerging from this collection is the transition from traditional single-biomarker analyses toward multi-factorial and biologically informed prediction systems. Several studies constructed prognostic signatures using high-dimensional omics datasets derived from The Cancer Genome Atlas (TCGA), Gene Expression Omnibus (GEO), GTEx, and other public databases. Advanced statistical approaches, including LASSO regression, Cox proportional hazards modeling and machine learning algorithms, were widely employed to identify clinically relevant molecular signatures.

Among these contributions, multiple studies focused on the development of gene-based prognostic models for diverse malignancies. For example, prognostic signatures derived from palmitoylation-related genes, immune-associated genes, metabolism-related pathways, methylation profiles, and tumor microenvironment-associated biomarkers demonstrated strong predictive capabilities across ovarian cancer, pancreatic cancer, melanoma, prostate cancer, osteosarcoma, lymphoma, and other tumor types. Several studies further integrated immune infiltration analyses, immune checkpoint evaluation, and drug sensitivity prediction, thereby extending molecular prognostic models into clinically actionable therapeutic contexts.

Another prominent aspect of this Research Topic is the increasing integration of tumor immune microenvironment analysis into predictive oncology. Multiple studies investigated immune infiltration landscapes, inflammatory signaling pathways, cytokine-associated signatures, and tumor–immune interactions to improve prognostic stratification and therapeutic prediction. These findings are particularly relevant in the era of immunotherapy, where precise characterization of tumor immune status has become critical for treatment selection and personalized management strategies.

The collection also reflects the growing adoption of interpretable and explainable AI methodologies in cancer prediction modeling. While machine learning and deep learning algorithms have shown substantial predictive power, clinical implementation requires transparent and biologically interpretable frameworks. Several studies within this Research Topic addressed this challenge by combining high predictive performance with clinically understandable nomograms, visualization platforms, and explainable modeling strategies. The incorporation of web-based calculators, user-friendly prediction interfaces, and calibration analyses further highlights the increasing focus on clinical applicability and translational usability.

In addition to omics-driven models, several articles explored multimodal prediction systems integrating imaging, radiomics, and clinicopathological data. Imaging-assisted predictive frameworks demonstrated promising applications for risk stratification, treatment response evaluation, and disease progression assessment. These multimodal approaches exemplify the broader movement toward comprehensive precision oncology systems capable of integrating heterogeneous data sources into unified clinical decision-support tools.

Importantly, the studies included in this collection collectively emphasize that predictive modeling in oncology is increasingly evolving beyond purely statistical exercises toward biologically grounded and clinically deployable frameworks. Many studies incorporated external validation cohorts, independent datasets, decision curve analyses, and receiver operating characteristic (ROC) analyses to strengthen robustness and generalizability. Such efforts represent an important step toward bridging the gap between computational discovery and real-world clinical implementation.

Despite these advances, several recurring challenges are also evident throughout the collection. Many predictive models remain retrospective in nature and rely heavily on publicly available datasets, which may introduce population bias, batch effects, and limitations in reproducibility. In addition, heterogeneity in sequencing platforms, preprocessing pipelines, and analytical methodologies continue to complicate cross-study standardization and external applicability.

Another major challenge concerns the limited availability of large-scale multicenter prospective validation. Although numerous studies demonstrated promising predictive performance in retrospective cohorts, relatively few models have undergone rigorous prospective evaluation in diverse patient populations. Future translational efforts will require closer collaboration among computational scientists, clinicians and biostatisticians to ensure that prediction models are reproducible, interpretable, and clinically meaningful.

Furthermore, as predictive architecture becomes increasingly sophisticated, balancing predictive accuracy with transparency and interpretability remains essential. Excessively complex models may hinder clinical trust and limit practical implementation despite strong computational performance. Consequently, explainable AI, standardized reporting frameworks, and clinically interpretable modeling strategies will likely become increasingly important in future oncology research.

Looking forward, several emerging technologies are expected to further accelerate the development of next-generation clinical prediction systems in oncology. Advances in single-cell sequencing, spatial transcriptomics, liquid biopsy technologies, federated learning, multimodal AI systems, and large-scale real-world clinical databases may enable the construction of increasingly dynamic and individualized predictive frameworks. Simultaneously, the integration of imaging analytics, digital pathology, and molecular profiling into unified computational pipelines may further enhance precision oncology and personalized treatment decision-making.

Overall, the studies included in this Research Topic provide a comprehensive overview of the current landscape of bioinformatics-driven clinical prediction modeling in cancer. Collectively, they illustrate the remarkable potential of integrating multi-omics data, computational biology, machine learning, and clinical information to improve prognostic assessment and therapeutic decision-making. At the same time, the collection underscores the ongoing need for validation, interpretability, standardization, and clinical translation. We hope that this Research Topic will serve as a valuable resource for researchers and clinicians working at the intersection of oncology, bioinformatics, and AI, while also fostering future interdisciplinary collaborations that advance precision cancer medicine.

